# Adherence challenges and opportunities for optimizing care through enhanced adherence counseling for adolescents with suspected HIV treatment failure in Kenya

**DOI:** 10.1186/s12913-022-08373-9

**Published:** 2022-07-29

**Authors:** Michelle M. Gill, James N. Ndimbii, Rose Otieno-Masaba, Millicent Ouma, Stella Jabuto, Boniface Ochanda

**Affiliations:** 1 Elizabeth Glaser Pediatric AIDS Foundation, Washington DC, USA; 2 Elizabeth Glaser Pediatric AIDS Foundation, Nairobi, Kenya; 3 Centers for Disease Control and Prevention, Center for Global Health, Division of Global HIV & TB, Kisumu, Kenya

**Keywords:** HIV, Children, Adolescents, Viral load, Adherence, Counseling, Kenya

## Abstract

**Background:**

Adolescents living with HIV (ALHIV) experience higher mortality and are more likely to have poor antiretroviral therapy (ART) adherence and unsuppressed viral load (VL) compared to adults. Enhanced adherence counseling (EAC) is a client-centered counseling strategy that aims to identify and address barriers to optimal ART use and can be tailored to the unique needs of adolescents. This study aimed to better understand adherence barriers among ALHIV with suspected treatment failure and their experience with EAC to inform future programming.

**Methods:**

A qualitative study was conducted in Homa Bay and Turkana counties, Kenya in 2019 with adolescents and caregivers of children and adolescents living with HIV with suspected treatment failure after ≥6 months on ART and who had received ≥1 EAC sessions. Sixteen focus group discussions (FGDs) were conducted; five FGDs each were held with adolescents 12–14 years (*n* = 48) and 15–19 years (*n* = 36). Caregivers (*n* = 52) participated in six FGDs. Additionally, 17 healthcare workers providing pediatric/adolescent HIV services participated in in-depth interviews. Audio recordings were transcribed and translated from Kiswahili or Dholuo into English and coded using MAXQDA software. Data were thematically analyzed by participant group.

**Results:**

Participants identified adolescents’ fear of being stigmatized due to their HIV status and their relationship with and level of support provided by caregivers. This underpinned and often undermined adolescents’ ART-taking behavior and progress towards more independent medication management. Adolescents were generally satisfied with EAC and perceived it to be important in improving adherence and reducing VL. However, problems were noted with facility-based, individual EAC counseling, including judgmental attitudes of providers and difficulties traveling to and keeping EAC clinic appointments. Participant-suggested improvements to EAC included peer support groups in addition to individual counseling, allowing for greater flexibility in the timing and location of sessions and greater caregiver involvement.

**Conclusions:**

The findings provide opportunities to better tailor EAC interventions to promote improved ALHIV adherence and caregiver-supported disease management. Multi-prong EAC interventions that include peer-led and community approaches and target adolescent and caregiver treatment literacy may improve EAC delivery, address issues contributing to poor adherence, and position adolescents to achieve viral suppression.

**Trial registration:**

ClinicalTrials.gov: NCT04915469.

## Background

Children and adolescents in sub-Saharan Africa comprised 90% of youth living with HIV in 2020 [1, 2]. Kenya has one of the largest populations of people under 19 years old living with HIV globally, including 99,000 adolescents (10–19 years) living with HIV in 2020; there were also 1500 AIDS-related deaths among adolescents [3]. HIV is the national leading cause of death for all ages [4]. In 2020, adolescents and young people ages 15–29 years accounted for 61% of all new infections in Kenya among people ≥15 years [5].

Adolescents living with HIV (ALHIV) experience higher mortality and poorer health outcomes than adults living with HIV. They are less likely to initiate antiretroviral therapy (ART) and be retained in care, and more likely to have poor ART adherence and an unsuppressed viral load (VL) [6–10]. In 2018–2019, viral load suppression among people living with HIV ≥15 years was about 72% compared to about 48% in children and adolescents < 15 years living with HIV in Kenya; the viral suppression rate was approximately 61% among adolescents 10–19 years only [11]. A review of 2012–2016 data also from Kenya demonstrated VL of ≥1000 copies/mL was more common in adolescents 10 - < 20 years (36.6%) compared to 13.3% for adults 30 - < 60 years [12]. Given the high HIV incidence among adolescents and young people, helping ALHIV adhere to ART is critical for their own health and to reduce the risks of antiretroviral drug resistance mutations and HIV transmission to sexual partners.

Enhanced adherence counseling (EAC) is a targeted client counseling strategy that aims to identify and address barriers to ART adherence to facilitate viral suppression. Aligned with World Health Organization recommendations, the Kenyan 2018 ART guidelines recommend that clients with a VL test result of ≥1000 copies/ml after six or more months of ART receive a minimum of three EAC sessions within a three-month period as a way of identifying and addressing adherence-related factors contributing to potential treatment failure [13]. This was based on a 2013 systematic review indicating that 70.5% of patients re-suppressed on the same ART regimen after EAC. Adolescent-targeted EAC is aligned with the national 2014 Adolescent Package of Care (APoC), as they both aim to address the clinical, psychosocial and structural needs of ALHIV. While EAC is focused on adherence, through assessing cognitive, behavioral, emotional and socio-economic barriers, mental health screening and developing an adherence plan to address identified barriers, APoC includes broader components, such as clinical assessment, communication and counseling, mental health, nutrition, sexual and reproductive health, psychosocial support and adolescent transition to adult care and treatment, but does not specifically address adherence [14]. However, EAC content and delivery is not standardized across, or even within, countries and more recent literature indicates it may not work as well for ALHIV. A 2019 systematic review and meta-analysis estimated that only 46.1% of patients with an initially elevated VL were able to re-suppress after EAC, with lower re-suppression rates among children (31.2%) and adolescents (40.4%) compared to adults (50.9%) [15]. Studies in Lesotho, Eswatini, and Uganda also found low rates of viral suppression or re-suppression following EAC in children and adolescents [16–18].

While EAC programming presents an opportunity to achieve higher rates of treatment adherence and viral suppression among ALHIV, it must be tailored to their unique challenges. Documented barriers to treatment adherence for adolescents include issues around disclosure, stigma, discrimination, school, transitioning out of pediatric care, treatment side effects, awareness of one’s own HIV status, and limited HIV knowledge [19–24]. Adherence programs targeting this group must address these context-specific barriers to achieve increased rates of ART adherence and viral suppression.

## Methods

The primary aim of this study was to better understand the issues affecting adherence among ALHIV with suspected treatment failure and their experience with EAC to inform future interventions that are tailored and optimized for effectiveness.

### Study setting

The study was conducted in Homa Bay and Turkana counties in Kenya. Homa Bay has an HIV prevalence of 19.6% among people 15 years and above and bears the largest burden of HIV in Kenya, while Turkana has a prevalence of 6.8%; county-specific estimates for younger age bands were not reported [11]. The study took place in five facilities across the two counties with the highest numbers of VL tests among clients < 19 years performed between October 2016 and June 2017. EAC was implemented broadly across Kenya, including in study counties, in 2019. Components as described under APoC were introduced in Homa Bay County in 2016, but not until late 2019 in Turkana. Both counties also implemented other adolescent-focused services to address adherence, viral suppression and other issues among younger clients, such as psychosocial support groups (PSSG). These groups were open to all adolescents, regardless of viral suppression or EAC status, and while groups had established membership rosters, some were not functional or operating with regularity at the time of data collection.

### Enhanced adherence counseling description

As outlined in the national guidelines, upon suspected treatment failure (VL ≥ 1000 copies/ml), child and adolescent clients and their caregivers underwent a minimum of three EAC sessions [13]. Sessions were meant to be scheduled 2 weeks apart; while this could depend on individual client needs, ideally, they were to be completed within 3 months. A repeat VL was to be performed within this timeframe following the high VL result and with good adherence, to determine if a regimen switch was necessary. Adherence was assessed based on pill count, compliance with appointment schedule and score on a standardized medication adherence scale. Providers were to assess adherence and review the individualized adherence plan at each EAC visit. EAC was often provided by adherence counselors or peer educators, but all provider cadres were trained on EAC as part of the guidelines and administered EAC as needed, including clinical officers given their role prescribing ART. EAC sessions covered topics such as implications of and possible reasons for high VL, barriers to adherence, development of an adherence plan, the need for repeat VL testing, and the role of the client/caregiver and health facility staff in HIV disease management. Compared to EAC standard of care, the standardized package represented a more consistent operationalization of EAC, with better alignment to the guidelines, including development of EAC standard operating procedures (SOPs), training of providers on psychosocial support and communicating with children, adolescents, and their caregivers using the SOPs, mentorship to providers and individualized case management, in addition to provider-led EAC sessions.

### Study design, population, and sampling

Data were collected as part of a larger pre-post intervention study assessing uptake and clinical outcomes of a standardized EAC package delivered to children and adolescents aged 0–19 years with suspected treatment failure who were accessing HIV care in the two counties. The main study assessed differences between the EAC package under standard of care and standardized versions. The results presented here are from the qualitative evaluation only. Focus group discussions (FGDs) were conducted with adolescents 12–19 years and caregivers of children and adolescents living with HIV. Caregivers were recruited if they had a child < 10 years; however, many reported experiences with their adolescent children as well, so their responses are included in the analysis when it could be reasonably inferred that they were referencing their older children. Separate FGDs were conducted with participants who received either the initial standard of care or standardized version of EAC. However, as few differences in responses emerged between those receiving the different versions, the qualitative findings are presented for both groups together, while highlighting a few significant distinctions. In-depth interviews (IDIs) were also conducted with healthcare workers (HCWs) and there were no distinctions made in the interviews regarding the different versions of the EAC intervention.

Support group leaders and HCWs used a recruitment script to inform potential FGD participants about the study. Research assistants then provided more information to those who were willing to take part. They obtained verbal informed consent from participants ≥18 years and emancipated minors; verbal informed consent and assent were obtained respectively, from parents and non-emancipated adolescents 12–17 years. Emancipated minors were defined as heads of households, married adolescents, or those with children. Verbal consent was obtained, because otherwise, the written consent document would be the only record linking participants to the study and the principal risk would be the potential harm resulting from a breach of confidentiality.

HCWs were recruited by the research assistants and underwent verbal informed consent before any study activities were initiated. They included providers who had served in a facility for at least 3 months and were directly involved in providing services, including EAC, to children and adolescents living with HIV.

### Data collection and analysis

Qualitative data was collected between August and October 2019. Research assistants used a semi-structured interview guide to conduct the qualitative sessions and collected demographics and HIV/ART-related history from participants using a brief close-ended form. FGDs were conducted in Kiswahili, Dholuo, or English, depending on participants’ preference. Sixteen FGDs were held in total; five with adolescents 12–14 years, five with adolescents 15–19 years, and six with caregivers of children and adolescents. FGDs for adolescents and caregivers were conducted in the same four of the five study facilities. Two FGDs of adolescents 12–14 years were conducted at one health center; two FGDs of adolescents 15–19 years were held at the other health center. Each of the two county referral hospitals held FGDs representing both adolescent age bands. Adolescent participants from the same PSSG at facilities were recruited to be in the same FGDs so they did not have to disclose their HIV status to others. All seventeen IDIs were conducted in English with HCWs.

FGDs and IDIs were audio-recorded, transcribed, and translated to English (as needed) by research assistants. The transcripts were then coded using MAXQDA 2020 (VERBI Software, 2019). Based on the interview guides, an initial codebook with codes and definitions organized around key themes was developed and shared among the researchers. The codebook was revised after reviewing and coding the first several transcripts. Following coding by two study team members, reports were generated for each code and then analyzed using matrices and narrative summaries by participant group (adolescents, caregivers, and HCWs and EAC package (standard of care and standardized). A raw summary report was developed and further refined to describe patterns in the data and address study objectives.

### Ethical approval

The study received ethical approval, including a waiver of written informed consent, from the University of Nairobi/Kenyatta National Hospital Ethics Review Board and Advarra Institutional Review Board in the United States.

## Results

Adolescent participants (*n* = 84) had a mean age of 14.6 years (SD: 2.0) (Table 1). Females comprised 47% (*n* = 39) of the study population. Nearly all adolescents (*n* = 82, 98%) were in school. Participants had been on ART for a mean duration of 7.6 years (SD: 2.9); 20% were able to correctly identify their ART regimen. Of the adolescents, 45% had disclosed their HIV status to at least one person and the majority (79%) were living with their parents. Fifty-two caregivers participated in interviews, with 92% (*n* = 48) female, though age and relationship to child are missing for one FGD (*n* = 8) whose children received the standard of care EAC version in Turkana County. Caregivers with available data (*n* = 44) had a mean age of 38 years (SD: 11.4); 64% (*n* = 28/44) were the child’s biological mother (data not shown). Seventeen (33%) caregivers of children receiving the standard of care EAC participated in two FGDs; 35 (67%) caregivers of children receiving standardized EAC participated in four FGDs. Cadres of HCWs interviewed (*n* = 17) included six clinical officers, four adherence counselors, six peer educators, and one adherence nurse. They all provided services to ALHIV and indicated that there was an adolescent-specific clinic at their facility.

**Table 1:** Demographics and background information of adolescents participating in FGDs

Variable	***N*** = 84
**Gender,** female, n (%)	39 (47%)
**Age at the time of discussion**, n (%)	
*Mean (standard deviation [SD])*	14.6 (2.0)
12–14 years	48 (57%)
15–19 years	36 (43%)
**County of residence**	
Homa Bay	67 (80%)
Turkana	17 (20%)
**EAC package version**	
Standard of care (6 FGDs)	33 (39%)
Standardized (4 FGDs)	51 (61%)
**If currently attending school, highest level of school achieved**, n (%)	
Some primary school	63 (75%)
Some/completed secondary school	18 (22%)
Any post-secondary school	1 (1%)
Does not attend school	2 (2%)
**Duration on ART, in years**, mean, SD	7.6 (2.9)
**Able to name ART regimen**, n (%)	17 (20%)
**Disclosed HIV status to anyone**, n (%)	38 (45%)
**Currently live with**, n (%)	
Parents	66 (79%)
Grandparent(s)	9 (11%)
Other family	8 (10%)
Other, not family	1 (1%)

### Cross-cutting issues affecting adherence

#### Stigma and discrimination

Adolescents and caregivers named a multitude of barriers to adherence, including arriving at home late in the evening and missing the drug-taking time, being away at boarding school, having visitors in the home or traveling outside one’s own home. The underlying theme of these responses was a reluctance to take their pills around others and inadvertently disclose their HIV status, fearing stigma and discrimination. If the time was missed, there was some confusion from both adolescents and caregivers about whether or not they should take their drugs late or skip altogether. Because of these fears, adolescents often did not carry drugs with them; a few who did described using alternative containers for their pills or mixing different drugs together to hide that they were antiretrovirals or so they would not rattle and make noise. Adolescent-specific days allowed them to attend clinic without the presence of adult clients. However, some adolescents reported being fearful that neighbors may see them in transit to clinic or that they will be seen entering the clinic, in the waiting room or in other common areas. A few adolescents reported postponing their appointment to avoid meeting someone they know.

Adolescents attending boarding school, more commonly found in the 15–19-year groups, presented a unique set of challenges. From adolescent and caregiver respondents, these included fear of stigmatization from classmates if seen taking pills, unsupportive teaching staff who were asked to store drugs and allow adolescents to attend clinic for appointments, and longer than anticipated exams or classes preventing them from exiting to privately take their medication on schedule.

*“So you see, timing is just a problem … for those who are in boarding school. They are just disadvantaged because there are some schools you don’t even trust your own self, like the school I was in. You can take [drugs] to the nurse or whatever, but the moment you will step out of that room … rumors will be everywhere. So you have to take a risk of taking that drug … So you see there is a problem with stigmatization in high schools.” (Adolescent 15-19Y).*

Several providers also raised the issue of adolescents not wanting their peers or others at the place they are boarding to see them taking drugs. They concurred with adolescent reports of gossiping and name-calling. Stigma and peer pressure were also adherence barriers commonly reported by caregivers.

#### Caregiver-child interactions

The dynamic between caregivers and their children was the most frequently reported barrier to adolescent adherence by caregivers. Caregivers, such as this female respondent, recounted their frustration with still having to remind their older adolescent children to take their drugs.

*“For him to deteriorate, is because he does not wake up and take drugs at 7am … he is 18 years, hence an adult. Do you think such ages are supposed to be pleaded with to take drugs? You ask him if he has taken and says yes. But since he woke up, he has not gotten out of his bed. He has not for sure. Such character contributes to taking drugs incorrectly.” (Caregiver).*

Some caregivers described trying to foster independence in their older adolescents and being met with children lying that they had taken their medication. A few caregivers were concerned that their children would not take medication in their absence which limited their own activities. Adolescents, including those 15–19 years of age, confirmed that they relied on others, typically mothers, to remind them when it was time to take their pills. Only a few adolescents described taking their drugs on time by themselves.

Other caregivers acknowledged their failure to remind their child to take their drugs or provide support given their own work demands or other stressors. Sending children out for errands or chores causing them to arrive home late was noted only twice among caregivers, but was frequently reported by adolescents. A couple other caregivers noted that these tasks could contribute to poor adherence in their children. Adolescents also reported other issues reflecting a lack of perceived caregiver support. This included not having food prepared with which to take one’s drugs, being locked out of the house and unable to get to their pills, and more general conflict that may not be related to HIV, but which discouraged them from adhering to their medication. Younger adolescents, as illustrated in the quote below, more frequently reported arguing and occasionally other forms of abuse by parents and other caregivers.

*“Sometimes the challenge comes from our parents on how they handle us and throw tantrums on us and you go without taking drugs.” (Adolescent 12-14Y).*

On the other hand, this caregiver described the importance of having a strong relationship with her child, which allowed her to be engaged in the process while her son exercised independence over his care.

*“You know when you are close to your child, you can find a way of getting easily to him. For example, my child, when he comes back from the hospital he has to tell me everything. What has been done to him, what is required and how everything was done...He knows his clinic appointment dates and can never miss.” (Caregiver).*

HCWs considered support from home as instrumental in attaining viral suppression, especially caregivers providing reminders or supervision when taking medication. However, the most frequently cited barrier among HCWs involved caregiver/child issues. They noted that frequent changes to the named caregiver or caregivers who were non-biological, HIV-negative, preoccupied, or had limited communication skills could contribute to poor adolescent adherence. Moreover, caregivers who shifted medication responsibility onto adolescents without providing adequate support and who were less engaged in their child’s drug-taking were more likely to have an adolescent with a high VL.

*“Okay … you find that most of the adolescents who have high viral loads, they are being left alone to take pills without close supervision. So the adolescent, they pretend to come to take pills, and they pretend to be swallowing but they are not taking. So in the long run, when the VL is out, the caregiver says her child is taking pills.” (Adherence counselor).*

### Enhanced adherence counseling

#### EAC perceived importance and acceptability

Adolescents and a couple caregivers described the importance of EAC for adolescents. This was largely in terms of receiving advice on adherence, being better informed on why VL increases, the importance and effects of ART, and being encouraged to adhere well to their medication, by becoming aware of their VL levels or to avoid negative consequences. Messages included that one could never stop taking treatment, even if they felt healthy. One adolescent in the standardized EAC group said explicitly EAC was not important. EAC was also often mentioned as an opportunity to learn about pill-timing and how to integrate drug-taking into daily schedules, while still meeting the demands of school and other responsibilities. How to properly store and travel with drugs was also raised a few times.

*“The good thing we find in that room is that it helps us with advice. When you have high viral load you will be asked what time you take drugs or you go to school so that you can be helped.” (Adolescent 12-14Y).*

Only a handful of adolescents described EAC as an outlet for exploring or addressing psychosocial and other factors that may affect their ability to adhere to treatment and their overall well-being; one explained that the provider offered them a space to speak openly about their issues. A provider said asking his clients for the reasons why one’s VL has increased allows for their inner reflection. A few other adolescents described that EAC encouraged them to address stigma, particularly to encourage them to overcome it in order to take drugs while traveling or in school. When asked what counseling does for adolescents with high VL, one adolescent responded that it can,

“*… help remind or help you correct your ways as to why your viral load is high. And another one to help those who are stigmatized to get out of that bond. And then another one is to make the family or maybe the people around you, that probably might have been giving you problems during your drug-taking, to understand why you are taking that drug, and to take action or maybe be reminded why you should take your drugs.” (Adolescent 15-19Y).*

Only two adolescents cited EAC as an opportunity to engage family in one’s medication management. This included helping family members to understand why the adolescent was taking drugs as well as how to better support the adolescent in their adherence. For their part, caregivers often described the education and motivation provided through EAC by providers as one of the keys to improving their children’s adherence. Caregivers often viewed their role as encouraging or bringing their children to clinic to attend EAC and reinforcing counseling messages at home. Only a few caregivers described a direct role in EAC sessions, but it was not clear if they were referring to their older or younger children.

*“As [a] parent, we can also talk to them back at home encouraging them to come to the doctor for enhanced adherence counselling.” (Caregiver).*

#### Challenges to EAC uptake and completion

While many respondents had positive impressions of EAC services, adolescents, particularly in the standard of care FGDs, cited harsh or judgmental HCWs as a challenge with EAC most frequently. They often felt as if EAC providers were quarreling with them or scolding them. Adolescents and a few caregivers on behalf of their children reported that EAC sessions had instilled fear in them.

“*Nothing can give me courage to go to that room … especially that doctor, I never used to like him. He would talk rudely to people, talking to people the way he wants … I never liked him. So there is nothing that can make me go back there again.” (Adolescent 15-19Y).*

*“You know I can be a counselor and you’re a client, then I tell you I am tired of you, you don’t suppress and I have been talking to you every day and that is not a good approach. A child is someone you just [need to] know how to approach, you have to be at the same level, [that] is when the child can tell you … ‘that’s why I miss drugs or do not take drugs’, but if you’re quarreling, then the child won’t come back to you.” (Peer educator).*

Adolescents noted that once they had missed one session, they did not want to go back because they were afraid providers would be angrier than usual. Complaints were also raised from a few female adolescents about sexual harassment from older male providers.

Several adolescents and some caregivers bringing children to sessions identified the challenge of visit frequency, the inconvenience of many trips to the clinic in a short period of time. This was reportedly exacerbated by the financial burden of transport fare, particularly if adolescents were traveling long distances to the clinic. A few adolescents also described the sessions as too long and repetitive.

*“That counseling is basically just the repetition of what you have already been told during pre-counseling or maybe post counseling. So you see, most of the people are like, ‘Oh! They are just going to repeat the same things I know.’” (Adolescent 15-19Y).*

*“Sometimes you go to the hospital and you are found with high viral load so you’re called to be talked to and you take a long time, then go back home late. So next time when such a thing happens, you might fail to come for enhanced adherence counseling.” (Adolescent 12-14Y).*

### Participant recommendations to improve EAC uptake

#### Improving interactions with providers and caregivers

The most cited recommendation primarily from adolescents was for providers to be friendlier.

*“Sometimes, you can come for the first time and the one who is in there, talks to you nicely and treats you nicely. The next time you will want to come back.” (Adolescent 15-19Y).*

Suggestions were also given about how to make interactions more positive. An example of this was to frame the discussion in a way that avoids blame and instead focuses on how the adolescent can improve.

*“It is the work of the providers to tell the adolescent to adhere well to medication … the provider should just tell you that this month your VL has gone high … so check where you went wrong and make changes … instead of  saying that … you have stopped taking your drugs … with some bad voice like that one.” (Adolescent 15-19Y).*

*“So I think what can benefit them is to tell them, having high viral load doesn’t mean that you have committed a crime … if we can negotiate and try to see which are the factors that made you not to adhere to medication, if you can rectify them, you can still be LDL [undetectable]. So, the perception they normally have, they normally term EAC as you are going to court. So, they have a negative attitude towards EAC. Because normally we try to question them on factors that contributed to their high viral load … in most of the cases, most of them don’t like those questions.” (Clinician).*

Some HCWs themselves stressed the importance of a multi-disciplinary team who could support adolescents, convey friendly attitudes to establish trust and rapport, and be understanding of the conditions and hardships that affect their disease management and overall well-being.

*“Are we providing adolescent-friendly services that can make these people feel at home? You see the mind of the adolescent. Do we have people who understand them? For example, they come and like, they are in a world of their own … are we in a position of understanding and getting better ways of talking to them, giving them proper information at the right time?” (Clinician).*

On the other hand, about one-third of HCWs recommended more involvement from caregivers to support their children with high VL. This would allow caregivers to understand the importance of EAC and discuss the reasons why drugs are not being taken and be empowered to remind the child to take their drugs, provide informed monitoring of their child at home and even perform directly observed therapy as needed.

*“What I suggest is we can use treatment literacy sessions and this [would] specifically involve the caregiver, can be the mother or the relative of the child, to enhance good adherence.” (Adherence Counselor).*

This counselor continued that the caregiver should be further involved in adherence action plan development to reduce their VL. A couple HCWs noted that caregivers do not consistently attend EAC sessions with the adolescents in their care; one caregiver said even if she attended clinic with her adolescent, she did not accompany him into EAC rooms, as they are considered to be adults. As above, caregivers, along with a few adolescents, suggested that caregivers should encourage attendance at sessions and reinforce counseling messages at home, but they did not echo the HCW recommendation to attend EAC together.

#### Changing the structure or location of EAC sessions

Looking across all participant groups (in four FGD each with adolescents and caregivers and in the majority of provider interviews), the most frequently made recommendation to changing the session structure and facilitation was to create EAC-specific peer groups in addition to individual EAC counseling. This recommendation was raised more often in the standardized EAC adolescent and caregiver FGD. While a few caregivers were concerned this might lead to adolescents sharing misinformation, and suggested ensuring clinicians were present as well, far more adolescents and caregivers felt peers could learn from each other and it might make them feel less alone.

*“I think the first EAC is one-on-one. You explain your problems and also try to find a solution but the second EAC is just going to remind the person. So … if for example, we are undergoing second EAC, we should be put together and everyone can talk about their problems and what they did so that others can learn from them instead of talking to individuals.” (Adolescent 15-19Y).*

HCWs frequently mentioned peer groups as a means to facilitate adherence directly and indirectly through providing bonding activities with other adolescents, supporting transition to adult care, and addressing stigma and other psychosocial issues. While many respondents touted the benefits of adolescents with high VL coming together to share challenges and feel less alone, a couple HCWs and a couple caregivers felt that virally suppressed adolescents should share their ideas for adherence management with peers experiencing high VL and encourage them to achieve viral suppression. Caregivers and adolescents repeatedly mentioned that it would be useful to reduce the number of sessions, while some HCWs were open to extending time between EAC sessions from 2 weeks to 1 month. Other recommendations were made to address the logistical challenges with attending clinic on a more frequent schedule for the duration of EAC. These included providing transport fare, holding sessions at alternative locations, such as having counselors visit adolescents at home, and providing counseling by phone or at their schools; these suggestions were less frequently mentioned by HCWs.

## Discussion

This study presents adherence challenges of adolescents and caregivers of children and adolescents with suspected treatment failure and their experiences with an EAC intervention that aimed to help address these challenges. The fear of being stigmatized or discriminated against as a result of their HIV status, as well as their interactions and level of support received from caregivers, underpinned and often undermined adolescents’ drug-taking behavior and progress towards more independent medication management. Adolescent experiences and perspectives on EAC, often echoed by caregivers and HCWs, suggested that these issues are not being comprehensively addressed in sessions and that there is an opportunity to better tailor EAC interventions to promote improved adherence and caregiver support and medication management among ALHIV. Adolescents and caregivers were generally satisfied with EAC and perceived it to be important in improving adherence and reducing VL. However, problems were noted with facility-based, individual EAC counseling, including judgmental attitudes of providers and difficulties traveling to and keeping EAC clinic appointments. Participant-suggested improvements to EAC included peer support groups for ALHIV with unsuppressed VL in addition to individual EAC counseling, allowing for greater flexibility in the timing and location of sessions and greater caregiver involvement.

While stigma and fear of discrimination emerged as a significant barrier to adherence, only a few adolescents reported that this was addressed in their counseling sessions. HIV-related stigma, in its different forms, has been shown to be a persistent and powerful barrier to adherence and retention among adolescents [21–23, 25–28]. Hickson & Mayers described adolescents and young adults’ fear of rejection and internalized shame due to an HIV diagnosis and purported that simple transference of knowledge about HIV and treatment had less of an influence on adherence than a sense of belonging and being accepted and supported in one’s social groups [25]. Stigma, including its intersection with the school setting, and lack or presence of support also emerged as key factors influencing adherence among youth 12–19 years living with HIV in South Africa and Botswana [27, 29]. Peer support groups were perceived not only to promote adherence, but also to cultivate adolescent independence, providing a supportive setting to express themselves, respite from stigma and discrimination, and an opportunity to establish social connections with peers confronted with similar issues [28]. Finally, adolescent girls and young women living with HIV in Zambia struggled with anticipated stigma that was mediated by emotional support from close family members and helped them achieve good adherence, though they still expressed a desire to have more peer interactions [26]. In our study, peer support groups were recommended across all participant groups to be included as part of the EAC package. This could help to address stigma and other factors contributing to poor adherence, including for ALHIV attending boarding school reporting heightened experiences of discrimination.

Studies assessing the effectiveness of adolescent/child-focused EAC programming have found poorer virological outcomes when compared with adults [15, 18], though contextual evidence on these gaps is limited. Our qualitative study found that two major challenges were provider attitudes and the frequency of additional clinic visits, resulting in repetitive session content and exacerbating challenges with accessing services (e.g., paying for transport fare, school conflicts). These challenges mirror health facility factors affecting clinic attendance and adherence among children and adolescents that have been well-established in the literature, such as securing transport, sub-optimal quality counseling, and poor attitudes of hospital staff [30–32].

While the content of the sessions may differ, the EAC intervention followed the same facility-based model for routine HIV care and treatment services. Given the challenges that resulted in adolescents requiring EAC in the first place, these findings suggest that alternative models of care may help to improve EAC uptake and completion, particularly those that could address the cross-cutting issues of stigma and caregiver support. For example, youth peer mentors living with HIV who provided active role modeling and problem-solving significantly decreased internalized stigma and increased viral suppression rates among pediatric clients [33]. A multi-pronged intervention in Zimbabwe included intensive targeting for adolescents with low virologic or immunological profiles. The intervention was comprised of facility and home visits plus daily phone contact from adolescent treatment supporters paired with a nurse as well as separate support groups for adolescents and their caregivers. These adolescents experienced a 42% lower prevalence of high VL or death at nearly 2 years post-intervention and improved adherence management [34]. Caregivers also exhibited improved treatment literacy, with adolescents reporting a ‘more sympathetic household environment.’

Our study showed that even older adolescents were reliant on their caregivers to ensure they took their drugs, which stirred resentment among some caregivers. On their part, some adolescents felt they were not being fully supported by caregivers, for instance by not prioritizing their drug-taking over other demands or activities. Failure to promote adolescent autonomy can be a barrier to transition, setting them up for future challenges when they will be responsible for their own care [35]. However, adolescents need to be adequately supported by caregivers as they gradually become more autonomous in areas such as appointment and medication management; rapid changes could only worsen adolescent struggles with adherence [20, 35]. Improving both caregiver and adolescent treatment literacy can support this process, with EAC sessions that involve high quality counseling and friendly providers, in which caregivers accompany their children, but the children and their needs and concerns are the focus of the sessions [20, 30, 32]. There was limited data to support that caregivers attended EAC sessions with their children, though some caregivers in our study described how they could facilitate adolescent EAC by being supportive at home and reinforcing messaging. However, this is more likely to be successfully achieved if caregivers are present to receive the same counseling messages and treatment literacy, including around medication timing and the handling of missed/late doses, and are directly involved in adherence planning. Treatment literacy embedded in an EAC approach also offers an opportunity to integrate “undetectable = untransmittable” messaging to enhance viral suppression, treatment continuity as well as mitigate self-stigma, while promoting healthy behaviors among adolescents [36]. Reframing the counseling sessions more positively, away from blaming adolescents for high VL or poor adherence and identifying opportunities for improvement was raised by respondents in our study. Adolescents on treatment in Malawi described their appreciation for positive reinforcement and incentivizing of pill-taking; negative provider or caregiver feedback or actions, which can prompt medication refusal or missed doses (and lying about missing doses for fear of punishment), can create a cycle of poor disease management [32].

This study has some limitations. Use of IDIs and FGDs have the potential for desirability bias in an effort to tell interviewers what they want to hear. Interviewer training focused on establishing rapport with participants to put them at ease by developing the interviewers’ ability to ask and elicit responses in a non-judgmental and non-leading way, ultimately to help mitigate this bias. Secondly, this study focused on behavioral and other non-biomedical explanations for suspected treatment failure. It should be acknowledged that drug resistance could have also contributed to the high VL of adolescents in this study. The prevalence of drug resistance has been shown to be high among youth on first-and second-line ART with virological failure [37–39]. Finally, there were gaps in the data collection among caregiver respondents. Conducting additional FGDs in the standard of care group may have allowed for better detection of differences by EAC version. In addition, we neglected to capture their children’s ages as part of the demographic data. While we recruited caregivers who had children < 10 years, many reported on their experiences with their adolescent children, though we do not know how many children nor their ages to provide better context to their responses. To address this weakness, we only included caregiver data in the paper that we could be relatively certain was pertaining to their older children (e.g., use of the term *adolescent*, including the adolescent’s age in the response). Despite these limitations, this study is unique in that it describes the adherence challenges and experience of EAC through the lens of adolescents with high VL (as well as their caregivers) and points to ways to help achieve optimal adherence that could be more fully addressed in EAC and HIV programming for ALHIV.

## Conclusions

This study expands on the existing evidence base of adolescent adherence with a focus on the cross-cutting issues of stigma and caregiver interactions affecting those with high VL and the extent to which an EAC intervention addresses these barriers. While there was overall satisfaction with EAC among adolescents, we found that building upon a similar individual counseling, clinic-based model offered in routine care, on a more frequent schedule mandated by the EAC intervention, may have limited EAC uptake and completion. Multi-pronged EAC interventions that include peer-led and community approaches, more constructive provider interactions and targeted adolescent and caregiver treatment literacy may improve EAC delivery, effectively address issues like stigma contributing to poor adherence, and position adolescents to achieve viral suppression.

## Data Availability

The datasets generated and/or analysed during the current study are not publicly available as no quantitative datasets were used except for the descriptive demographic data. These data are available from the corresponding author on reasonable request.

## Abbreviations

ALHIV: Adolescents living with HIV
ART: Antiretroviral therapy
EAC: Enhanced adherence counseling
FGDs: Focus group discussions
HCW: Healthcare worker
IDI: In-depth interview
SD: Standard deviation
VL: Viral load
